# Role of Electroconvulsive Therapy in Major Depressive Disorder with Borderline Personality Disorder: Case Report and Literature Review

**DOI:** 10.7759/cureus.3211

**Published:** 2018-08-27

**Authors:** Saad Wasiq, Ahmad R Khan, Amber E Faquih, Hina Saeed, Hafsa Mahmood

**Affiliations:** 1 Psychiatry, University of Health Sciences, Islamabad, PAK; 2 Psychiatry, University of North Dakota School of Medicine and Health Sciences, Fargo, USA; 3 Graduate, Dow University of Health Sciences, Karachi, PAK; 4 Psychiatry, Sindh Medical, Ontario, CAN

**Keywords:** major depressive disorder, bpd, ect, electroconvulsive therapy

## Abstract

Major depressive disorder (MDD) becomes difficult to treat when the patient has a comorbid personality disorder. For such patients, even a combination of psychotherapy and pharmacotherapy has been ineffective. Electroconvulsive therapy (ECT) has been the first line of therapy for the treatment-resistant depression. We used this mode of therapy for a patient who had MDD along with borderline personality disorder and had failed trials of multiple medications and psychotherapy. ECT was very successful in our patient.

## Introduction

Depression is one of the most common psychiatric condition; recently it has been labeled as the leading cause of the illness burden throughout the world [1]. Depression as an isolated disease can be treated with different types of pharmacological therapies including selective serotonin reuptake inhibitors, tricyclic antidepressants, and some other groups. These medicines have proven to be very effective. Comorbid psychiatric conditions especially personality disorders are very common along with depression; according to some studies the prevalence rate of personality disorders among the patients of major depressive disorder (MDD) ranges from 30% to 80% [2]. Patients with such comorbid personality disorders are usually treatment resistant to psychotherapy or pharmacotherapy alone [3]. The recommended therapy for all the treatment-resistant depression is the electroconvulsive therapy (ECT) [4]. There is very limited literature available on this topic, and very few prospective trials have been conducted to evaluate the effectiveness of ECT in MDD patients with comorbid personality disorder. One such trial in which the number of subjects was 20 showed lower remission rates in these patients [5]. Some old literature reports have shown that indicators of personality disturbance were negative predictors of ECT response, but the lack of standardized diagnostic and symptom assessments renders interpretation difficult as most of these studies were carried out before the publishing year of Diagnostic and Statistical Manual of Metal Disorders III (DSM III) [6-11]. In this uncertain situation where there is no proven effectiveness of ECT in patients with MDD with comorbid personality disorders, we tried ECT on a patient who had been suffering from MDD along with the borderline personality disorder (BPD) for the last 10 years.

## Case presentation

A 33-year-old woman was admitted to psychiatry inpatient with a complaint of suicidal ideation. The patient has a past history of multiple psychiatric disorders like BPD, MDD, and anxiety issues for about last eight to 10 years. She had multiple suicide attempts in the past most recent being two weeks back when she tried to suffocate herself with the help of a medical device tubing. On inquiry, she said she just wanted to feel the pain, not kill herself. On further questioning, she was found to have passive suicidal thoughts as well as an active plan to harm herself. Her plan was to kill herself with the carbon monoxide poisoning by turning on the engines of four cars parked in the garage. According to her, she felt better at the time of the last admission and these suicidal thoughts just returned two to three days back. She had multiple admissions and emergency department (ED) visits related to her psychiatric conditions as well as five suicidal attempts. During one of her admission when she took multiple tablets of Advil® (Pfizer, New York, USA) in an attempt to kill herself, she was evaluated for ECT by a psychiatrist but the decision was made in favor of dialectical behavior therapy (DBT) as they felt these symptoms are because of her BPD. According to the patient she has been compliant with the therapy that has helped her in coping day-to-day issues. The patient also confirmed that she has never recovered from these active and passive suicidal thoughts which have progressed to even worse state in the last four months.

 On further evaluation, the patient reported feelings of hopelessness and worthlessness most of the time along with a guilt of things for what she has done in the past. She also reported a decrease in sleep to about five hours per night along with difficulty in staying asleep and poor appetite and energy. Her concentration was normal. She continues to engage in her interests in reading and photography. The patient states she has been a "warrior" for years. She endorses a few prior panic attacks where she felt shaky, short of breath, and had chest pain. She could not recall how long they lasted or when her last episode was. Screening for mania, psychosis, and obsessive compulsive disorder was unremarkable. There was no reported history of alcohol, tobacco, or illicit drugs.

Minnesota multiphasic personality inventory-2 (MMPI-2) results suggested the presence of depression, anxiety, overall distress, and a personality disorder. All of these scores are in the moderate to severe ranges and are rather similar to one another. The psychologist during this visit interpreted that these results do not suggest that the personality disorder is the main factor driving her clinical presentation and that her presenting symptoms are due to MDD. She was recommended ECT as it was determined that these symptoms are due to resistant MDD.

The patient had a past history of multiple psychiatric drug trials in the last eight years which included medicines like fluoxetine, sertraline, venlafaxine, amitriptyline and even augmented therapy with antipsychotics was tried with aripiprazole and thyroxine which all proved ineffective in this patient. Considering her condition and beneficial outcomes in such a treatment-resistant patient, a trial of ECT was the consensual decision of all the panelist psychiatrists. The patient agreed to this mode of therapy.

The first session was done with the parameters mentioned in Table 1. 

**Table 1:** First session parameters.

Electroconvulsive therapy (Session # 1)	
Pulse width (msec)	0.30
Pulse frequency (Hz)	30
Stimulus duration (sec)	2.00
Stimulus charge (mC)	28.8

After the first session parameters were changed for the rest of the 12 sessions which are mentioned in Table 2.

**Table 2:** Parameters for rest of the sessions.

Electroconvulsive therapy (Session # 2 onwards)	
Pulse width (msec)	0.30
Pulse frequency (Hz)	50
Stimulus duration (sec)	4.50
Stimulus charge (mC)	108

A total of 16 sessions were conducted with a break after 13 sessions. The frequency of sessions was three per week for the first 10 sessions and then two sessions every week and last three sessions were conducted once a week. The patient was evaluated after every session and there was a remarkable improvement from the sixth session onwards. After 13 sessions there was a thorough evaluation and the patient reported improved mood and no active or passive suicidal ideations and she was discharged. She remained symptom-free for four to five months but then reported again with another suicide attempt. She was restarted on ECT, and 16 more sessions were conducted with the same frequency and same parameters.

On her recent visit, she endorsed a significant improvement in her depressive symptoms and denied active suicidal ideations. She also reported an improved quality of life.

## Discussion

Major depressive disorder with a comorbid personality disorder is a treatment dilemma for all the psychiatrists. The advancement in psychopharmacology has successfully treated depression. The most common personality disorder co-occurring with depression is BPD [12]. Their relationship has been under study for a long time now; one study has proved the likelihood of developing BPD in a patient with early onset depression [13]. A number of other studies have expressed the negative response of MDD along with personality disorders to conventional antidepressants [5]. ECT is a therapy which is not used as a first line for the patients with MDD rather it is usually used for the treatment of resistant patients. As these patients with comorbid personality disorders are difficult to treat, there is a possibility of these patients responding to this mode of therapy as per this hypothesis.

Our patient was suffering because of uncontrollable suicidal thoughts. Our treatment approach in the first place was to target the BPD traits with the help of DBT which was helpful in controlling some of the symptoms related to the BPD but the suicidal thoughts persisted. In the second phase of treatment, the target was to treat the MDD which by now has acquired the tag of ‘treatment resistant depression’ after failed trials of pharmacological agents. The ECT trial was the treatment plan which was very helpful in controlling symptoms. MDD with a comorbid personality disorder has very poor outcomes with the ECT therapy, and among the personality disorders, BPD has shown the most resistant patterns with poor symptomatic improvements and remission as compared to other personality disorders [5]. The severity of BPD is a stronger predictor of subsequent MDD episodes and the depression due to MDD can get better if BPD is managed first and this was found to be true for 60%-70% patients in a study, which can also explain good ECT response in our patient where DBT improved BPD symptoms [14].

We did a small literature review on MEDLINE to evaluate the effectiveness of ECT for the patients of MDD with BPD and the results of these reports were mixed. Results and all characteristics of these studies are summarized in Table 3.

**Table 3:** Literature review of relevant clinical trials. PD, personality disorder; HamD, Hamilton Rating Scale for Depression; BDI, Beck Depression Inventory; SCID, structured Interview for Diagnostic and Statistical Manual of Mental Disorders (DSM).

Study	Sample size	Depression assessment	PD confirmation	Results
Flint and Hill-Johnes 2008 [15]	12 patients with BPD	Clinical	Clinical	Subjective improvement with ECT therapy
Feske et al. 2004 [5]	20 BPD and 119 with other PD or without any PD	HamD	SCID	Poor response in patients with BPD as compared to other PD patients and with no PD
Sareen et al. 2000 [16]	45 patients (30 with BPD)	Clinical	Clinical	Lower acute response and early relapse in patients with BPD
Zimmer et al. 1986 [4]	10 patients ( 3 with BPD)	HamD	SIDP	Acute response with PD the same as non-PD patients, but PD patients had higher 6-mo relapses and HamD scores
Krammer 1982 [17]	5 patients with BPD	Clinical or BDI	Clinical	No improvement in two patients, one showed some acute improvement, two showed some improvement but early relapse
Walter and Rey 1997 [18]	16 adolescents with PD (11 borderline) and depression	Clinical	Clinical	PD patients had lesser ECT response

The response we observed in the patient was very significant and to the best of our knowledge, there is no reported case where such a significant change was observed in the patient. The available literature related to this issue is very limited and outdated. Large-scale trials are required to establish the fact about the significance of ECT in these patients.

## Conclusions

We can conclude from this case where a patient with MDD along with a comorbid personality disorder had no success with the conventional pharmacotherapy as well as behavior therapy. After repeated suicidal attempts we decided to use an unproven therapy, i.e. ECT which has previously shown mixed results. In our case, the patient showed a significant improvement. We suggest a large-scale trial to establish the beneficial outcomes of ECT for these patients.
